# Environmental friendly warp yarn coating from feather wastes with enhanced toughness and tenacity via thiol-ene click chemistry grafting modification

**DOI:** 10.1186/s40643-025-00838-z

**Published:** 2025-02-13

**Authors:** Xing Pan, Daen Qin, Kaili Song, Aixue Dong

**Affiliations:** 1 Key Laboratory of Intelligent Textile and Flexible Interconnection of Zhejiang Province, College of Textile Science and Engineering, International Institute of Silk, Zhejiang Sci-Tech University, Hangzhou, Zhejiang 310018 China; 2 Key Laboratory of Clean Dyeing and Finishing Technology of Zhejiang Province, Shaoxing University, Shaoxing, Zhejiang 312000 China; 3 Key Laboratory of Advanced Textile Materials and Manufacturing Technology, Ministry of Education, Zhejiang Sci-Tech University, Hangzhou, Zhejiang 310018 China; 4 Zheijiang Sci-Tech University Tongxiang Research Institute, Tongxiang, 345000 China

**Keywords:** Biodegradable wrap sizing, Click chemistry, Keratin, Biomass waste

## Abstract

The development of biodegradable and environmental friendly wrap sizing agent is beneficial for reducing the impact of textile desizing effluent on environment. In this work, green and biodegradable keratin sizing agent with enhanced toughness and tenacity was prepared by adjusting side chain tails structure via thiol-ene click chemistry grafting modification for warp yarn slashing against film thermal-embrittlement and fracture during weaving. Feather keratin from waste feather has become an attractive target to replace petroleum-based Poly (vinyl alcohol) sizes due to its easy film-forming ability, excellent adhesive property, biodegradability and low cost. However, keratin yarn coating showed brittleness and easily peeled off from weaving yarn which highly restrict its application. Thus, polymer branched side chain tails with a structure that similar to the wrap yarn was grafted onto keratin molecular chain by utilizing its distinctive thio group as anchor point by means of click chemistry. The results showed that grafting modification could obviously improve the adhesiveness of keratin sizing by the principle of similarity compatibility. The sizing performance, desizing performance as well as its degradability were comprehensively studied. Successful utilization of modified keratin sizes will bring opportunities for the green and sustainable development of textile industry.

**Graphical Abstract:**

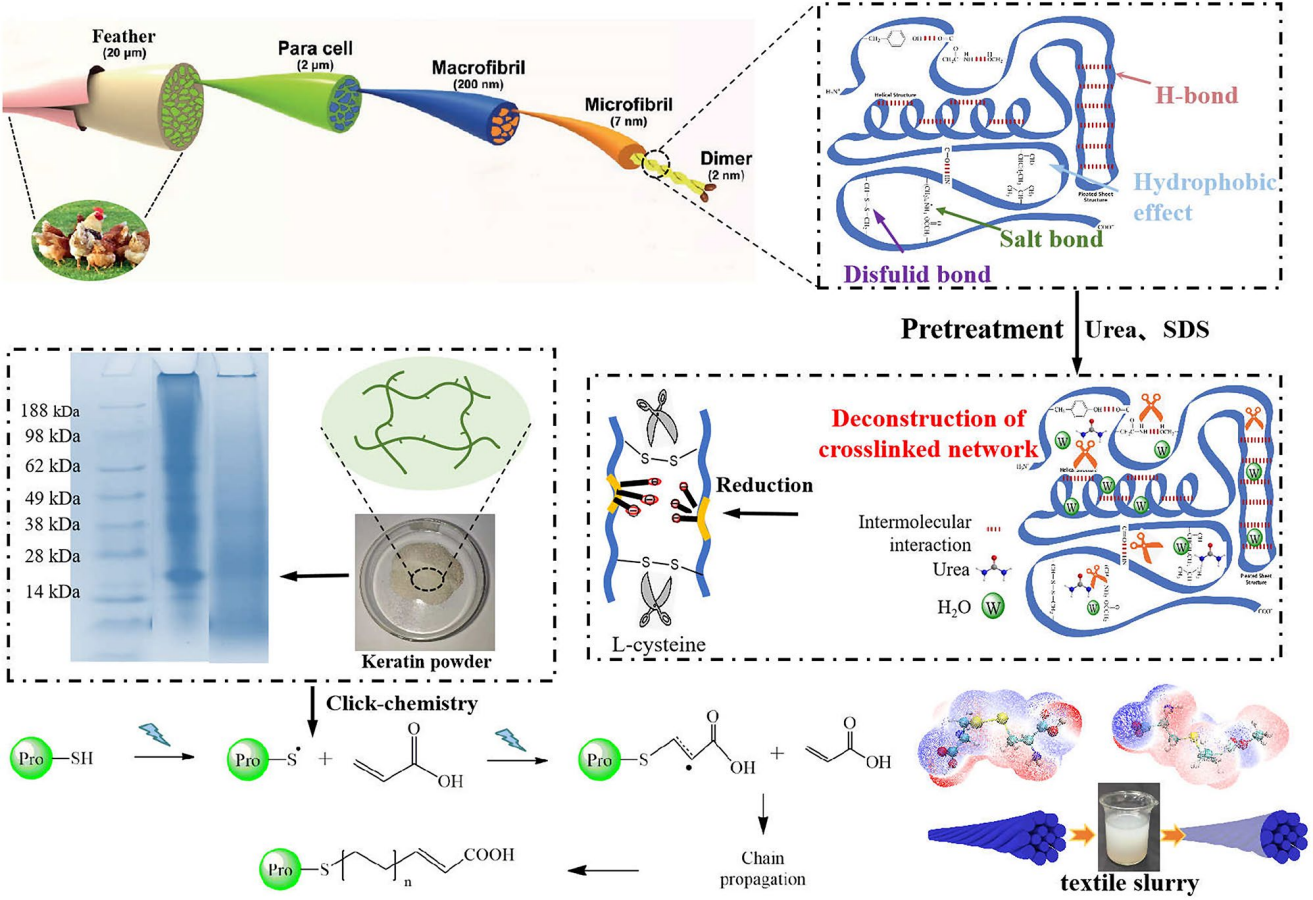

## Introduction

The green and sustainable development of textile industry is beneficial for environmental protection and carbon neutrality. As we all know, yarn sizing or slashing is an important and necessary procedure for the production of textile, which could endow better strength of wrap yarns and reduce its breakage during weaving. In addition, the size film that resulted on the surface of warp yarn could reduce the hairiness of yarns and increase its wear resistance via protecting warp yarn from abrasion during high speed weaving (Bolat et al. 2021). Thus, slashing act as an indispensable and important part of textile that could enhancing weave efficiency. Currently, petroleum based poly(vinyl alcohol), that is PVA, is the most abundantly used textile sizes as the result of its high film strength and toughness (Li et al. 2022a, b). However, the chemical structure of PVA is relatively stable. Thus, PVA could hardly be degraded by microbe and contributes about 40%of chemical oxygen demand (COD) in textile processing and causes serious environmental pollution (Shen et al. 2019). Therefore, it is urgent to exploit a biodegradable textile sizing agent with excellent sizing performance to replace PVA.

Excellent coating-film forming ability, satisfying adhesiveness to the sized yarns, easily desizing and biodegradable of the de-sized wastewater are the important and primary requirement for an ideal biodegradable textile sizing materials (Sarkodie et al. 2023). Currently, most frequently used green and biodegradable textile size to replace PVA are modified starch and water-soluble polyester sizes. Modified starch is biodegradable, but higher in price. Moreover, the weaving efficiency for starch sized high count and high density fabric could not compare with that of PVA (Li et al. 2021). Also, water-soluble polyester sizing agent belonging to a new type of environmental friendly textile wrap which in mainly applied in polyester yarns. Recently, plenty of researchers proved that regenerated protein from agricultural by-products or wastes such as wheat gluten, soy protein, keratin are suitable alternative to as biodegradable textile size to replace PVA (Zeng et al. 2023).

Among those protein sizing agents, regenerated keratin that extracted from waste feather was an ideal candidate for environmental sizing. Keratin is low cost and abundantly exist in waste feather from poultry industry which is usually disposed as solid waste, resulting in the occupation of valuable land. Comparing with other protein, keratin possessed higher content of disulfide bond which is beneficial for a better performance of sizing film (Pakdel et al. 2022). Therefore, utilizing and developing of keratin based textile sizes will favor the advance of biodegradable and green wrap sizing and sustainable development of global textile industry (Kim et al. 2022). However, the reported keratin sizing film is usually brittle which is mainly due to the breakdown of molecular chain during its dissolution and regeneration. Traditionally, keratin was extracted from feather wastes by alkali reduction process, resulted dramatically degradation of molecular chain and poor mechanical property of keratin sizing film. Besides, keratin sizing film could be easily peeled off from the surface of yarn especially under thermo-drying process as the result of its poor adhesiveness at high temperature (Xing et al. 2021). Thus, we are aiming at improving the mechanical tenacity and adhesiveness of keratin sizing film in this research.

According to the similarity effect, the adhesiveness of sizing materials would be higher if there are similar side chain with the structure of the sized wrap yarns. Thus, introduce some side chain that possess similar functional group with textile yarn could be an ideal way to improve the adhesiveness of keratin sizes (Ni et al. 2023). In addition, grafting modification could preferably enhance the mechanical property of keratin sizing film. Considering the unique and outstanding content of disulfide bonds within keratin, thio-ene click chemistry was adopted to introduce the designed side chain onto keratin molecular (Mi et al. 2020; Ye et al. 2020). Hence, in this research, environmental friendly and sustainable textile sizing agent from feather wastes with enhanced toughness and tenacity was fabricated via thiol-ene click chemistry grafting modification. Interestingly, the films showed excellent tensile properties and adhesiveness. We studied the sizing performance, desizing efficiency and cost to explore its prospective application (Khumalo et al. 2022). The results demonstrated modified keratin size could be considered as a promising substitute for poor biodegradable PVA sizes.

## Experimental

### Material

Animal feathers (duck feathers) in this experiment was purchased from Hangzhou Yongfeng eiderdown Products Co. Ltd. Cotton yarn, cotton roving, cotton/polyester (65/35) yarn, and roving were supplied by Tongxiang Zhouquan Factory. Acrylic acid (AR) and methacrylic acid (AR), Tianjin Yongda Chemical Reagent Co. Ltd. Methyl acrylate (AR), Chengdu Kelon Chemical Factory; MMA: Methyl methacrylate (AR), Shanghai Jingchun Biochemical Technology Co. Ltd.; IA: Itaconic acid (AR), AA: Acrylic acid (AR), MA: methyl acrylate (AR), MAA: methacrylic acid (AR) and TrIS-HCL solution, Shanghai Maclean Biochemical Technology Co. Ltd.; L-cysteine (AR), Shanghai Aladdin Biochemical Technology Co. Ltd.; Tris (2-carboxyethyl) phosphine hydrochloride (AR), Shanghai Hao Hong Biomedical Technology Co. Ltd.

### Keratin extraction

Keratin was extracted from duck feathers via reduction method based on previous reports[18]. Feathers (30 g) were pre-swelling in 300 mL of aqueous solution containing urea (220 g) for 4 h then cysteine (3 g) was added into the solution at PH 10.5 and 70 ◦C to obtain keratin solution. Then the solution was centrifuged at 9000 rpm for 20 min to precipitate undissolved feather residues. Then keratin solution was dialyzed with distilled water (DI) using dialysis cassettes (7000 Da) for 3 days.

### Graft polymerization

TCEP reducing agent was dissolved in ethanol solution (1:1 V/V) and configured to 50 mmoL/L mother liquor. A certain amount of reducing solution was added to the three-mouth flask, and the pH value was adjusted to 7 with Na2CO3/NaHCO3 buffer solution (pH > 9). 0.5 g solid keratin was added to the three-mouth flask under the protection of nitrogen, and the reduction was carried out at 20 ℃ for 4 h. A certain amount of initiator (3 wt%) and graft monomer (wt% of keratin: 20, 40, 60, 80, 100, 120) were added to the reduced keratin solution under the protection of nitrogen, and the pH value of the system was adjusted to 7. The graft reaction was finished after a certain time at 20 ℃. The product was filtered and thoroughly cleaned with distilled water and ethanol. Finally, the product was freeze-dried and stored in a dryer.

### Measurement of grafting parameters

In order to describe the degree of grafting reaction, graft rate and graft efficiency are used. In this experiment, grafting rate and efficiency were calculated by the change of free sulfhydryl group content before and after grafting, as shown in Eqs. (1) and (2) below.

1 $$\:\text{G}\text{r}\text{a}\text{f}\text{t}\text{i}\text{n}\text{g}\:\text{r}\text{a}\text{t}\text{e}\:\left(\text{\%}\right)=\frac{\text{A}}{\text{B}}\times\:100$$

2 $$\:\text{G}\text{r}\text{a}\text{f}\text{t}\text{i}\text{n}\text{g}\:\text{e}\text{f}\text{f}\text{i}\text{c}\text{i}\text{e}\text{n}\text{c}\text{y}\:\left(\text{\%}\right)=\frac{\text{B}-\text{A}}{\text{C}}\times\:100$$

Where, A is the -SH content (mmoL/g) of post-graft keratin, B is the -SH content (mmoL/g) of pre-graft keratin, and C is the amount of co-grafted monomers (g).

### FTIR characterization

FTIR was used to verify the successful graft of the monomer onto keratin. keratin and graft modified keratin were characterized and the functional groups in keratin were determined. By comparing the ir spectra of keratin and graft modified keratin, the similarities and differences in structure were predicted and the graft modified keratin was confirmed to be successful.

### Measurement of adhesion to fibers

Keratin was dissolved in a sodium hydroxide solution with a pH of 9 to make a concentration of 7% and heated to 80℃. The yarns were sized in a sizing machine (Jiangyin Tongyuan Textile Machinery Co., Ltd.GA392).The roving was wound with a self-made wire frame and immersed in the diluted 90 °C slurry solution for 5 min. The yarns and ravings were pre-conditioned at RH65%, 20 °C for 24 h and then tested on a yarn strength tester YG61C according to GB/T 3916 with the gauge length of 500 mm and the stretching speed of 50 mm/min. The yarn hairiness tester YG172 and yarn wear-resisting tester MHY-07867 were used to test yarn hairiness and friction resistance. The morphology of cross sections of sized and unsized cotton yarns were observed using scanning electronic microscope (SEM, TM3000, Hitachi, Japan).

### Film casting and tensile properties testing

Keratin sizes with/without modification were casted onto PTFE-coated plate respectively to form a film, balanced in Under constant temperature and humidity for 24 h.Films were tested on a universal material testing machine(Shenzhen Phobs Instruments Co., Ltd., Dx-400).The clamping distance was set at 20 mm, the tensile rate was set at 10 mm/min, the test environment was 25 ℃±2 ℃, and the humidity was 65%±2%.

### Biodegradation performance

Desizing was accomplished by immersing the fabrics in water at the water-to-fabric weight ratio of 50:1 under 90 ℃ and 20 ℃ for 5 min. After treatment, the fabrics were dried in an oven at 70 ℃ for 2 h and their weight before and after desizing was calculated to desizing efficiency. The biodegradability of COD and BOD5 of keratin size was determined. COD is determined according to GB11914-89 standard. BOD5 is determined according to environmental standard HJ 505–2009.

### Quantum chemical calculations

In this paper, the structure optimization and frequency calculation of DES and 2cystine are performed based on density function theory (DFT) with the B3LYP/6-311G(d, p) basis set using GAUSSIAN 09 software. The optimal structure and the most stable conformational energy were obtained according to the energy minimization principle.

## Result and discussion

### Preparation of chicken feather keratin size

**Fig. 1:**
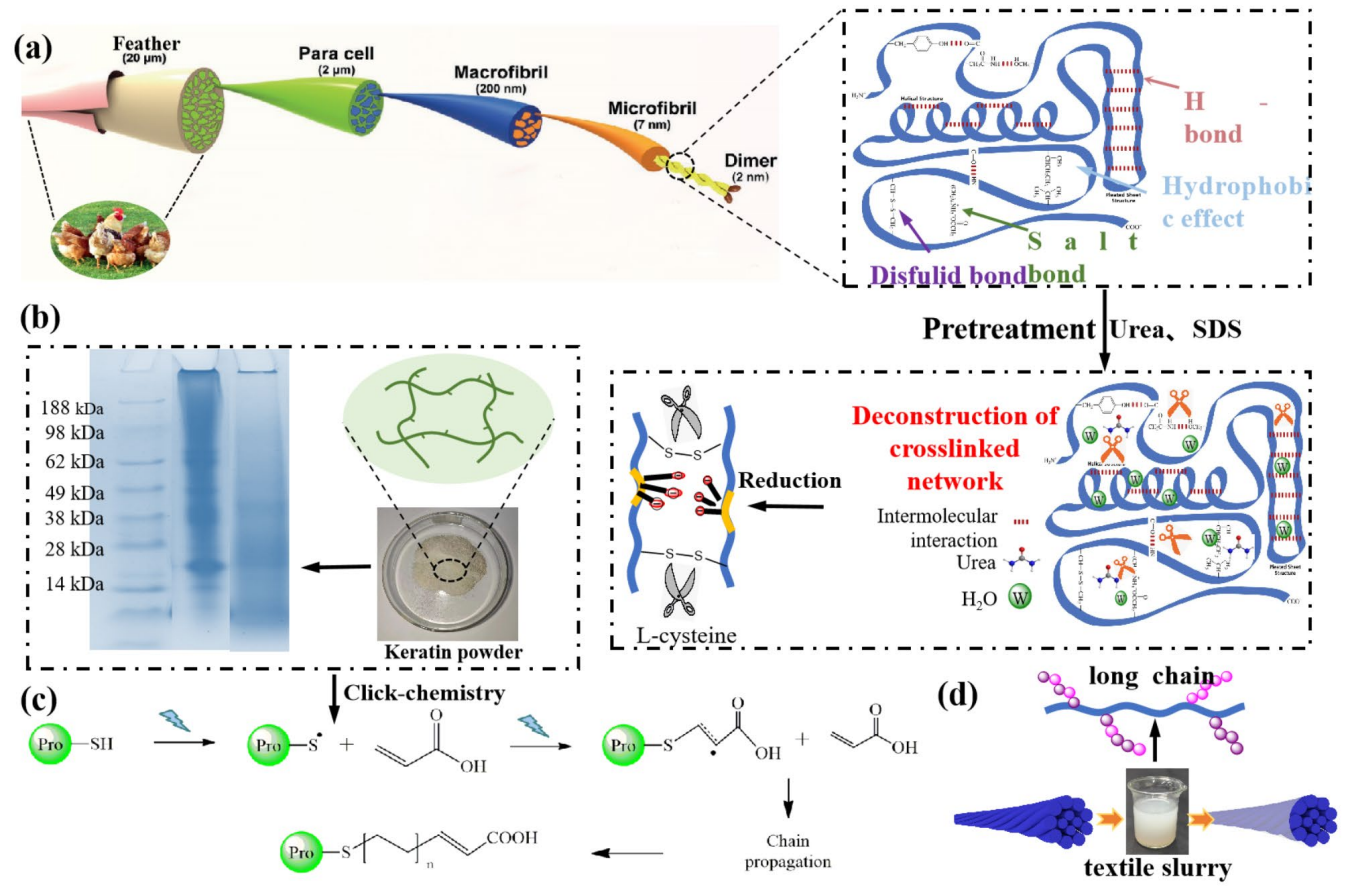
Schematic diagram of preparation keratin textile sizes. (**a**) keratin extracted by pretreatment assisted reduction method; (**b**) molecular weight of the obtained keratin; (**c**) “thiol-click” chemical grafting modified keratin; (**d**) schematic diagram of textile yarns before and after sizing

The hierarchical structures of chicken feather was shown in the Fig. 1(a). Commonly used method of keratin extraction is the alkali reduction method, that is the conventional reduction method. However, under the action of high temperature and strong alkali, the molecular chain of keratin would be broken, thus the molecular weight of the regenerated keratin is generally low and resulting in the poor mechanical properties of keratin sizing films (Kongdee Aldred et al. 2023). Therefore, in this research, we developed a pre-swelling followed reduction method with the purpose to obtain high molecular weight keratin via weakly deconstruction of keratin molecular chain. The pre-swelling is beneficial for the explosion of disulfide bonds and further reduce the alkalinity of the extraction system which could help to preserve the backbone of keratin molecular as shown in Fig. 1(b). The hydration ability of urea allows it to carry a large number of water molecules into the protein chains to swollen the chicken feathers and destroys the weak secondary bonds between keratin molecules (such as salt bonds, hydrophobic forces, and hydrogen bonds) and resulted the well exposure of disulfide bonds (Gunnarsson et al. 2021). As can be seen from Fig. 1 (b), keratin extracted by pre-swelling method showed strong molecular bond around 28, 38, 62KDa, while the molecular weight obtained by the conventional reduction method is around 28 KDa.

### Effect of monomer structure on the property of keratin sizing films

**Fig. 2:**
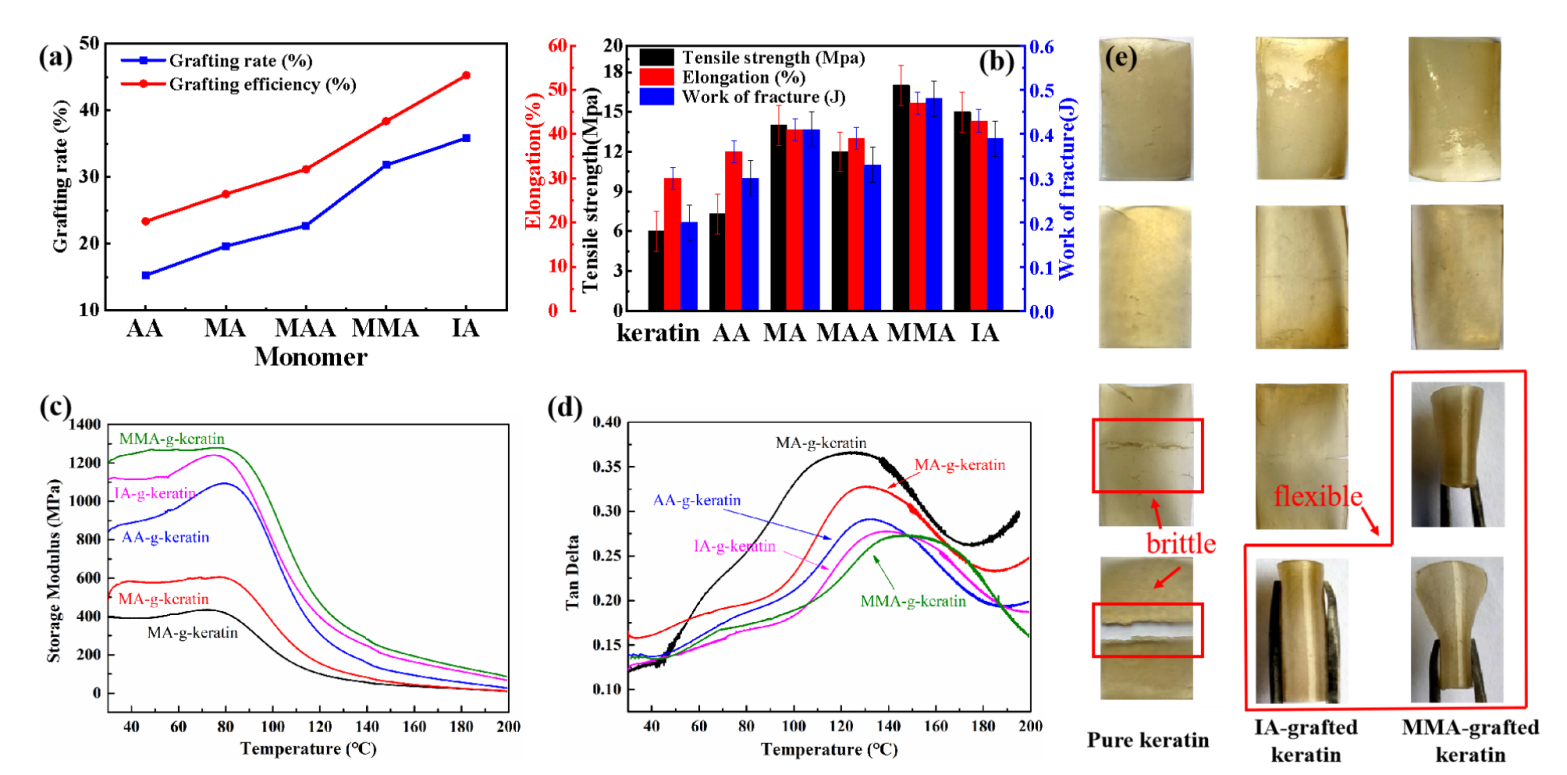
(**a**) Grafting rate and efficiency of different monomer; (**b**) The mechanical properties of grafted keratin sizing membrane; (**c**, **d**) DMA thermograms of storage modulus and damping factor of the grafted sizing membrane; (**e**) Image of the sizing membrane

The effects of different monomer is studied in detail on the properties of the regenerated keratin size films, including tensile strength, elongation and fracture work, as shown in Fig. 2. The grafting monomer is selected to be similar with the structure of the wrap yarns so as to improve the adhesion between keratin size and the yarns by utilizing the principle of proximity affinity (Zahara et al. 2021). As shown in Fig. 2 (a), the grafting rate and efficiency of MMA and IA are relatively high, compare with other selected monomer in this study. Figure 2 (b) shows that the tensile strength, elongation and fracture work of the modified keratin size films. The tensile strength of modified keratin increased significantly after grafting of different monomer and the best performance was obtained by MMA grafting modification. This may be because MMA and IA monomers with larger molecular weight are more likely to react with regenerated keratin than other monomers with smaller molecular weight that are easy to self-polymerize. The different monomers introduce increased molecular weight of the regenerated keratin and enhance the strength and toughness of the regenerated keratin size films (Chen et al. 2019). Among all grafted monomers, MMA and IA monomers with higher molecular weight, grafting rate and grafting efficiency enhance the strength and toughness of the regenerated keratin size films more significantly.

DMA is a versatile and effective tool to characterize the morphology, viscoelastic properties and thermo-properties of viscoelastic composite. In this research, DMA was employed for the purpose of investigating into thermo-mechanical property. Figure 2 (c) shows that the storage modulus of the regenerated keratin size films grafted with different monomers also varies with the increase of temperature, among which the storage modulus of the regenerated keratin grafted with MMA monomer is the highest. This may be because the carbonyl group on the grafted MMA monomer is more likely to be hydrogen bonded to the main chain of the keratin molecule (Xu et al. 2019). Then, the internal friction between the molecules is increased, showing a high energy storage modulus at the macro level, which prove that the keratin grafted with MMA monomer has a stronger breaking strength.

According to the damping coefficient tanθ, different monomers grafted with regenerated keratin have different damping coefficients. Figure 2 (d) shows that the tan delta is less than 1, which indicates that a good elastic complex cross-linking network is formed between the grafted monomer and the regenerated keratin molecules, which make the grafted regenerated keratin size films have good elasticity after the grafted monomer of regenerated keratin. Therefore, the macro performance is good toughness when stretched by external forces. Compared with other monomers, the damping coefficient of MMA monomer after grafting keratin regeneration is smaller, and the grafting effect is better (Rojas-Martínez et al. 2020). Figure 2 (e) shows the comparison of the properties of the regenerated keratin size films before and after grafting, as well as the comparison of toughness among different grafted monomers.

### Effect of monomer dosage on the tensile properties of keratin films

**Fig. 3:**
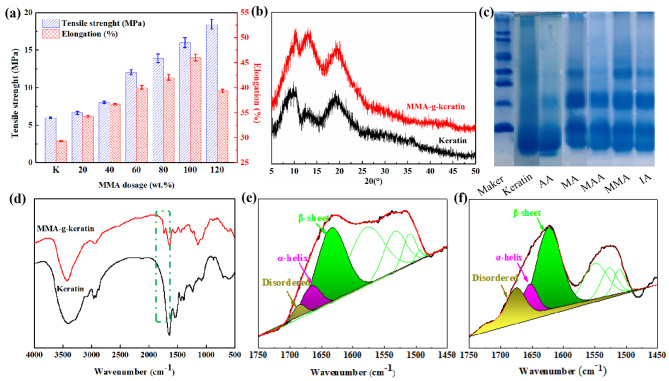
(**a**) Influence of MMA dosage on mechanical properties of keratin film, (**b**) XRD spectrum of keratin and MMA-g-keratin, (**c**) SDS-PAGE of the grafted keratin, (**d**) FTIR of keratin and the modified keratin, (**e** and **f**) peak fitting of amide I in FTIR spectrum

It is worthy noting that the dosage of monomer grafted onto keratin chain display significant effect on the molecular structure and tensile property of keratin sizing films. Thus, the crystalline structure and secondary morphology structure of the modified keratin were comprehensively studied as shown in Fig. 3. Figure 3 (d) shows the FTIR spectra of keratin and MMA grafted modified keratin. A new absorption peak of carboxyl stretching vibration is generated at 1740 cm^− 1^, which can prove that MMA (methyl methacrylate) monomer has been successfully grafted on the regenerated keratin molecular chain (Jiang et al. 2022).

Figure 3 (a) shows the effects of different amounts of MMA monomer on the strength and toughness of the regenerated keratin size film. With the increase of the amount of MMA monomer, the strength of the size film shows a gradual increase trend, and the elongation increases first and then decreases. This is mainly because the number of monomer molecules that can react in the system increases with the increase of the amount of MMA monomer. Then, more monomers are grafted onto the regenerated keratin molecules, which increases the molecular weight of the regenerated keratin size films (Zhang et al. 2019). Figure 3 (c) is the SDS-PAGE molecular weight characterization spectrum of all grafted regenerated keratin. It can be seen that the molecular weight bands of regenerated keratin in grafted MMA monomers are highly distributed and relatively large.

With the introduction of MMA monomer and the increase of dosage, the strength and toughness of the regenerated keratin size films are obviously enhanced. Figure 3 (b) shows that the peak strength and crystallinity of regenerated keratin after grafting MMA are higher than those of the original regenerated keratin without grafting modification. XRD analysis shows that the strength of the regenerated keratin size film after MMA graft modification is better. However, with the increase of the dosage to a certain value, MMA grafted on the side chain of regenerated keratin molecules will form a certain extent of encircling the regenerated keratin molecular chain in the spatial system. Figure 3 (e) (f) shows the results of infrared peak-splitting simulation of secondary structure composition of MMA grafted regenerated keratin size film. The peak fitting treatment of amide I band shows that the content of random curl conformation of MMA grafted regenerated keratin size film is less, which prove the strength and toughness of the regenerated keratin size film after MMA grafted modification (Li et al. 2022a, b).

### The sizing performance of modified keratin sizes

**Fig. 4:**
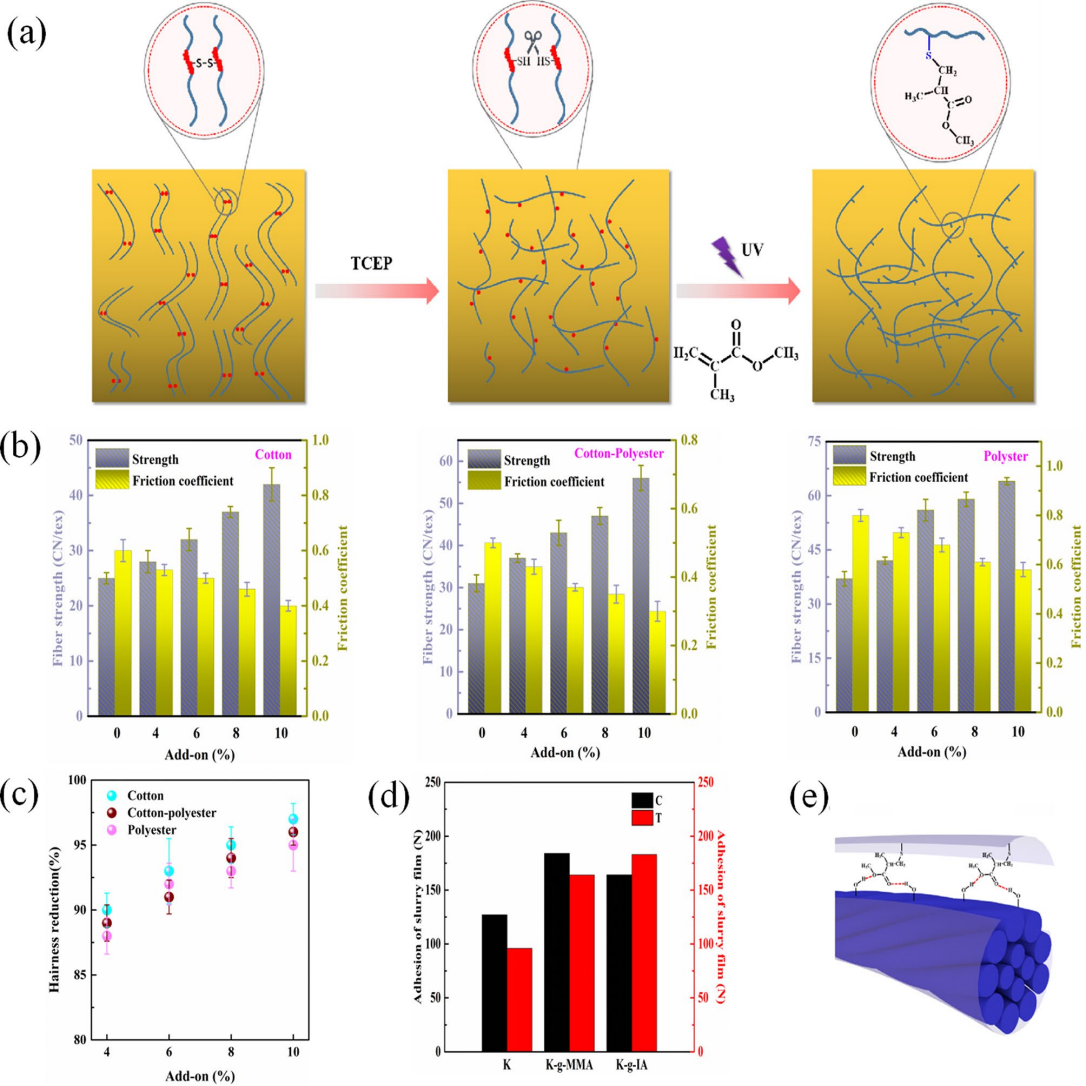
(**a**) schematic illustration of side chain of the modified keratin, (**b**) strength and friction coefficient of the sized yarns, (**c**) effect of add-on on the hairness reduction of the sized yarns, (**d**) effect of add-on on the adhesion of the size film, (**e**) adhesion interaction between sizes and the yarn

One purpose of sizing is to improve the tensile properties of warp yarns to reduce warp breakage during weaving and improve fabric quality. The grafted keratin size could better keep the warp yarn’s integrity since modified keratin sizing film could form a strong and tough cover layer on the surface of warp yarns (Jiang et al. 2019). In addition, long side chain which possess similar group as the yarns could be introduced onto keratin backbone and improve the adhesiveness of the modified keratin sizes as shown in Fig. 4 (a).

As shown in Fig. 4 (a), the adhesion between the regenerated keratin size and the polyester fiber can be improved by introducing long branched chains into the regenerated keratin molecular chain by grafting MMA with a structure similar to that of polyester fiber. Figure 4 (b) shows the relationship between the sizing rate of MMA grafted regenerated keratin size film in different types of yarns (cotton, polyester, poly-cotton blended yarns) and the strength and friction coefficient of the yarn after sizing. MMA grafted regenerated keratin size can enhance the adhesion to the yarn and improve the tensile property of the yarn, especially for cotton yarn. This may be due to the good hydrophilicity of cotton yarn and reclaimed keratin, the two are more close and the force is stronger (Li et al. 2020).

Figure 4 (c) (d) shows that when the sizing content is 10%, the wear resistance, wear resistance times and hair reduction rate of the three yarns are the optimal conditions, among which the sizing effect of polyester yarns is the most obvious and the wear resistance times are the highest. The wear resistance of the regenerated keratin size after graft modification is improved, which may be due to the protective effect of the regenerated keratin size film after graft modification on the size. The protective effect makes the keratin structure more stable and difficult to break, thus improving the wear resistance of the sizing yarn (Zha et al. 2021). The fiber hair reduction rate of the regenerated keratin size film increases after graft modification, which may be because the keratin with the molecular chain opened contains hydrophilic and hydrophobic groups, which have good adhesion to cotton and polyester yarns and are more able to attach surface hair so that the hair reduction rate of the modified keratin size film is enhanced (ÖZDEMIR 2018).

**Fig. 5:**
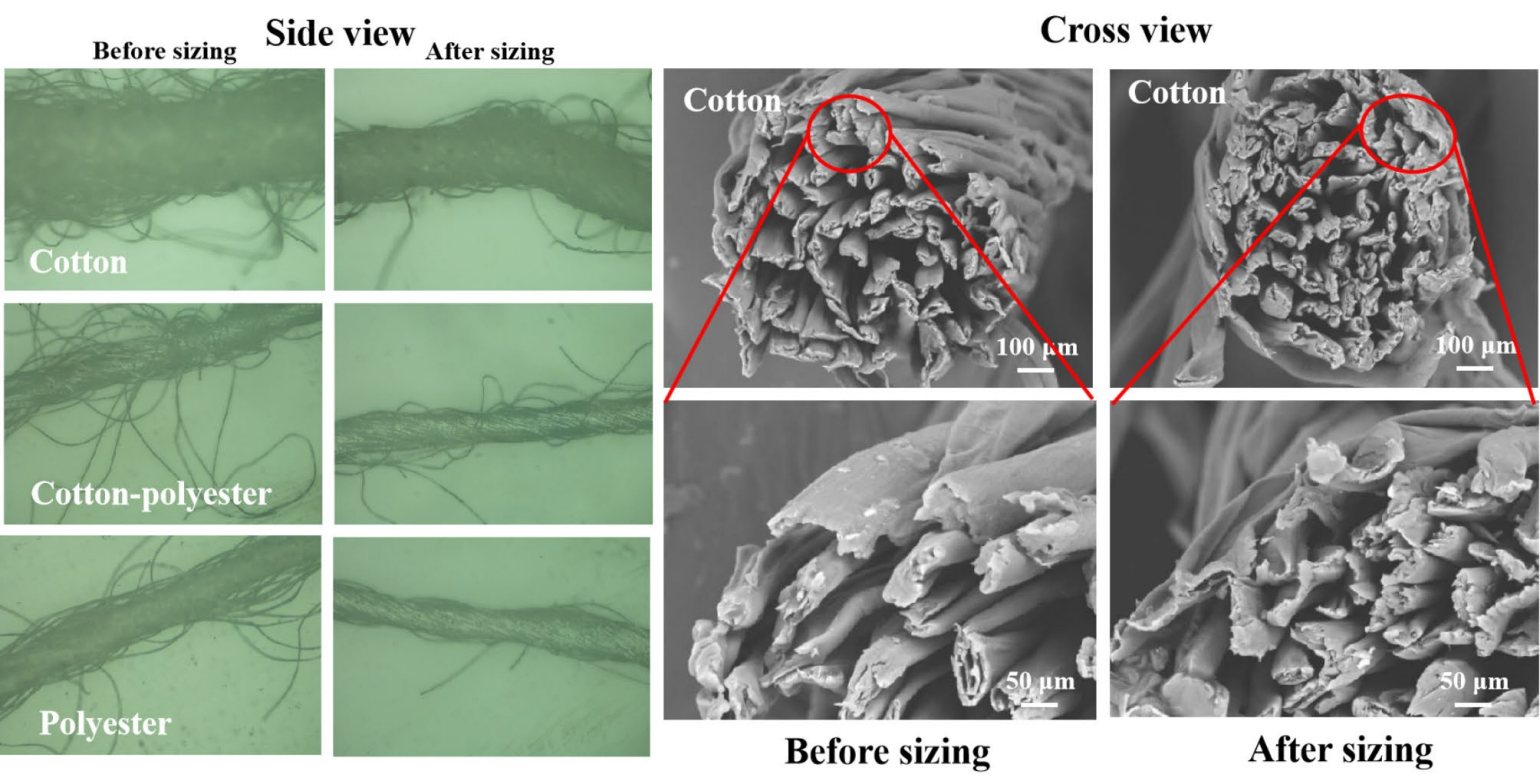
SEM morphology of the warp yarns before and after sizing

Figure 5 shows that the SEM cross view of cotton yarn. After cotton yarn sizing, the cross section becomes tighter, and the space between adjacent fibers is significantly reduced. The fibers of the yarn become tighter, which further enhances the strength of the yarn during weaving (Rafikov et al. 2020).

**Table 1:** The desizing efficiency of the sized warp yarns and BOD/COD of the desized wastewater

Type of fabrics	Desizing temperature/℃	Desizing efficiency/%	BOD_5_/COD
Cotton	90	95.1	0.54
Polyester	90	93.6	0.53
Cotton/polyester	90	94.8	0.56

After weaving, sizes should be removed from the fabrics in order to guarantee the dyeing and finishing quality. Thus, benign desizability is an indispensable quality for textile sizes. Table 1 shows the desizing performance of the modified regenerated keratin sizes. The desizing efficiency of keratin sized yarns are all higher than 92%, indicating that SPI based sizes possessed the quality of easy desizing. It is worth to mention that the BOD/COD of the desized wastewater are all higher than 0.5, which could be easily degraded in natural environment. The feasibility of desizing and the satisfying degradation behavior endow the green sizes prepared in this study a promising and sustainable sizes.

**Fig. 6:**
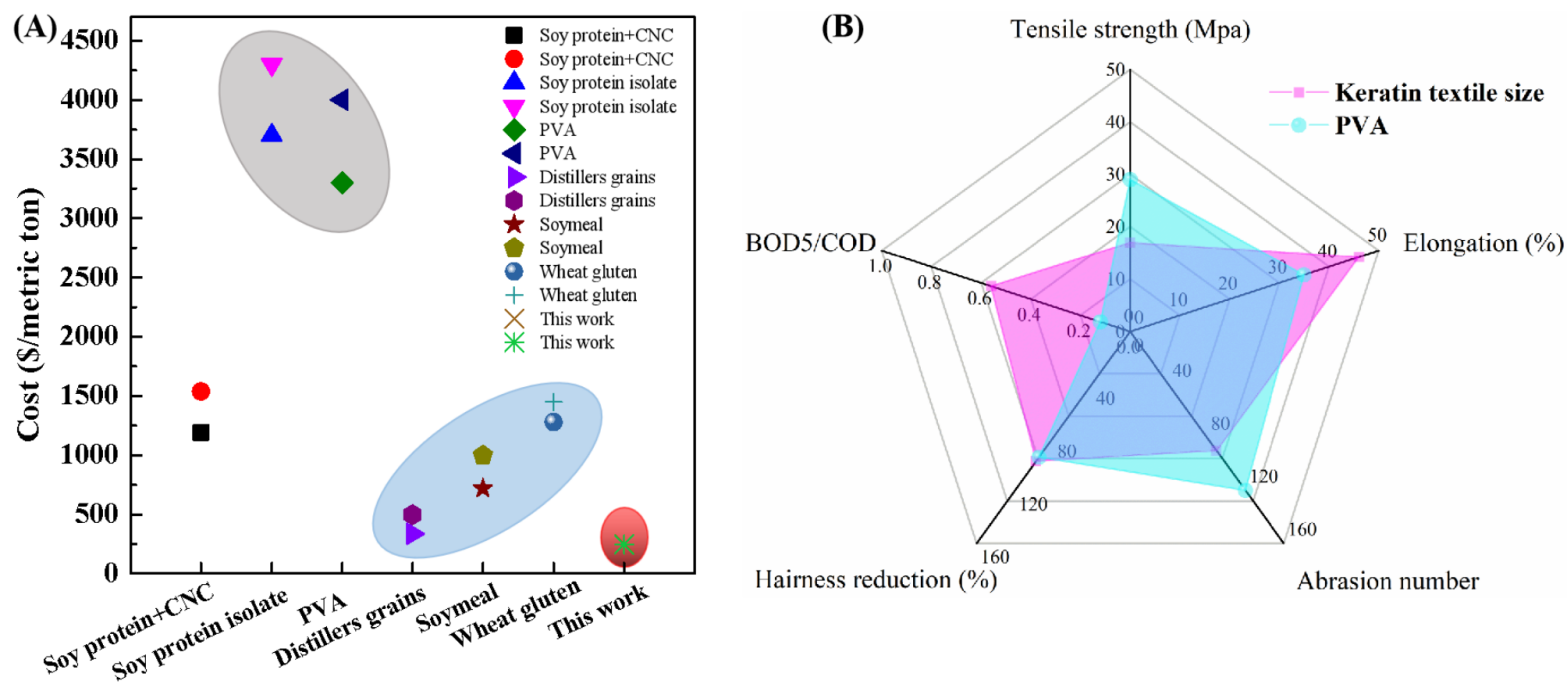
(1) Hairiness reduction rate of monomer modified cotton and polyester yarns; (2) Wear resistance of monomer modified cotton and polyester yarn

For industrial application, the cost of sizing agent is vital important factor determining its industrialization prospects. Thus, we also give a comprehensively evaluation of the cost of the prepared keratin sizes as shown in Fig. 6 (a). According to the optimized extraction conditions developed in this study, to get 1 ton of keratin size, 1.6 tons of feather should be used based on the yield of 62%.

Thus, cost of 1 ton of keratin size = cost of 1.6 tons of feather + cost of 15.3 ton of urea + cost of 0.08 ton of cysteine=$0.01*1.6+$15*15.3 + 1333*0.08=$336.1.

Hence, the cost of producing one ton of keratin size should be around $336.1. In this research, MMA is the optimized monomer of grating modification, and one ton of MMA-g-keratin size need 0.5 ton of MMA which cost around $50.

Thus, cost of 1 ton of modified keratin size = cost of 1 ton of keratin size + cost of 0.5 ton of MMA=$336.1+$50=$386.1.

Compared with the cost of commercial PVA ($2000-$2500/ton), MMA-g-keratin sizing agent is almost 75% lower in cost. Hence, using modified keratin sizing to substitute PVA in textile industry is both environmentally friendly and economically friendly. Also, we compared the cost of regenerated protein based sizing reported in literature in Fig. 6 (a), keratin based green sizes developed in this research share the lowest price (Zhao et al., 2019, 2023). In addition, we compared the sizing performance of MMA-g-keratin size with that of PVA in Fig. 6 (b).

### Mechanism of improved sizing performance

**Fig. 7:**
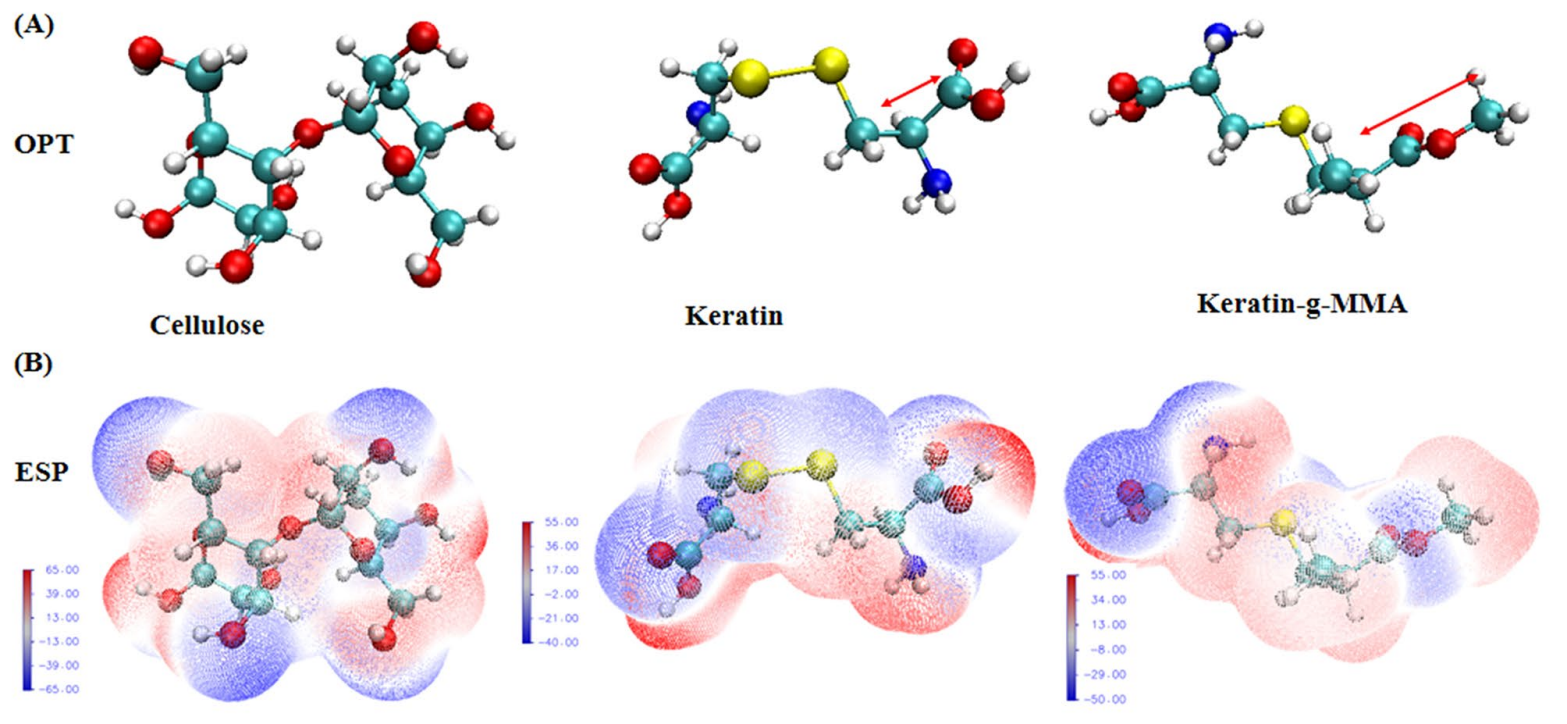
The optimized molecular structure of cellulose, keratin and MMA-g-keratin and their ESP map

In order to further elucidate the mechanism for the improvement of sizing performance from the point of molecular view, the optimized molecular structure of cellulose, keratin and MMA-g-keratin were analysis by Gaussian simulation as shown in Fig. 7. Glucose disaccharide was chosen as model compound for cellulose and cystine was chosen as model compound for keratin. The improved sizing performance is mainly due to higher adhesiveness between keratin and the wrap yarns which is the result of interaction force. As can be seen from, the difference in the interaction force between keratin and the yarns is mainly due to the change of side chain length. Figure 7 (a) presents the chain length of keratin and MMA-g-keratin, which is 1.56Å and 2.73Å respectively. In addition, the electrostatic potential of the side chain group in MMA-g-keratin exhibited more positive charge as shown in Fig. 7 (b). The surface of cellulose exhibited negative charge, which is the same as keratin. After grafted of MMA, the surface properties of modified keratin become more positive and the increase in the electrostatic attraction between the MMA-g-keratin and cellulose explain why the interaction forces between the modified size and yarns increase after grafting modification.

## Conclusion

In this work, green and biodegradable keratin sizing agent with favored toughness and tenacity as well as promising sizing and disizing property was prepared by adjusting side chain tails structure via thiol-ene click chemistry grafting modification. The structure of monomer on the degree of grafting, conformation of keratin and sizing performance of modified sizes were investigated and founded to exhibited enhanced tensile strength and elongation of the sizing films. Compare with keratin, MMA-g-keratin showed 156% and 50% incremental in tensile strength and elongation of the sizing film. In addition, the introducing of MMA as the side chain improved the adhesiveness of keratin size, which was expected to tackle with the thermo-brittleness problem of keratin sizes. The sizing performance, desizing performance as well as the degradability of modified keratin sizes are surprisingly satisfying, which could be an ideal alternative to PVA and promote sustainable and lowcarbon development of the textile industry.

## Data Availability

Data will be made available on request.
